# Laparoscopic adrenalectomy of pheochromocytoma following management of severe aortic stenosis with transcatheter aortic valve replacement under monitored anesthesia care sedation: a case report

**DOI:** 10.1186/s12871-023-01977-6

**Published:** 2023-01-11

**Authors:** Leon Yang, Lauren Hennis, Kevin Patel, Michael A. Saccocci

**Affiliations:** 1 Virginia Tech Carilion School of Medicine, Roanoke, VA USA; 2 Department of Surgery, Carilion Clinic, Roanoke, VA USA

**Keywords:** Aortic stenosis, Pheochromocytoma, Aortic valve replacement, Monitored anesthesia care, Case report

## Abstract

**Background:**

Management of a patient with an active pheochromocytoma and severe aortic stenosis remains controversial. Adrenalectomy for a pheochromocytoma poses a high risk for stroke, hypertensive emergency, and mortality, compounded by the cardiovascular instability of severe aortic stenosis. In this case report, successful management of an active pheochromocytoma with concomitant severe aortic stenosis was accomplished by performing transcatheter aortic valve replacement under monitored anesthesia care prior to laparoscopic adrenalectomy.

**Case presentation:**

An 84-year-old woman with severe aortic stenosis (valve area 0.53 cm^2^) presented with a symptomatic pheochromocytoma. Transcatheter aortic valve replacement was performed under monitored anesthesia care using a judicious approach of 100 mcg fentanyl total, 4 mg total of midazolam, and a background dexmedetomidine infusion. Alpha-blockade was maintained with 10 mg total of phentolamine mesylate. Laparoscopic adrenalectomy was performed after an uncomplicated postoperative course. The perioperative course for the adrenalectomy was unremarkable and the patient was hemodynamically stable. Postoperative course was uncomplicated and the patient was discharged from the hospital after 5 days.

**Conclusion:**

This case report demonstrated the successful approach of managing severe aortic stenosis through a transcatheter aortic valve replacement using monitored anesthesia care sedation prior to laparoscopic adrenalectomy of a symptomatic pheochromocytoma.

## Background

**Fig. 1:**
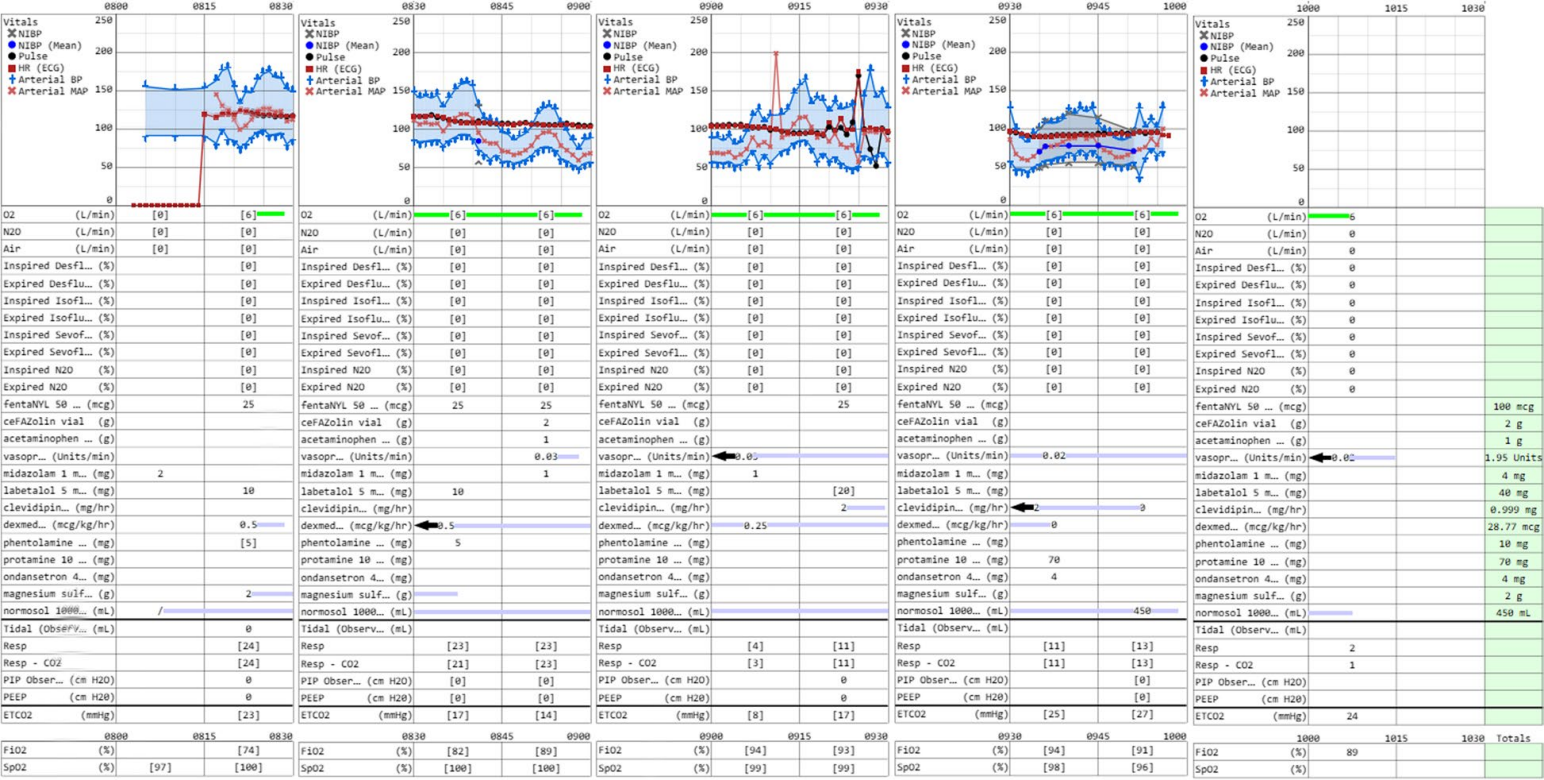
Intraoperative data during TAVR procedure

Transcatheter aortic valve replacement (TAVR) has become a safer alternative to open surgical aortic stenosis (AS) treatment in patients otherwise too high risk for traditional surgery due to serious comorbidities. As often seen in patients with severe aortic stenosis, insidious onset of disease ultimately leads to eventual progression to symptomatic effects and often mortality [1–4]. Pheochromocytomas, adrenal medullary tumors of chromaffin cells, produce robust amounts of catecholamines, and are classically associated with paroxysmal hypertension, tachycardia, and various other non-specific, insidious clinical manifestations. Alternatively, pheochromocytomas may be asymptomatic, alluding to their wide range of manifestations and difficulty in diagnosis (Fig. 1).

This case report shows a unique approach to managing AS in a patient with significant comorbidities, including a symptomatic pheochromocytoma and severe dementia, which also led to a limited traditional management of pheochromocytoma by adrenergic blockers. By performing a TAVR under monitored anesthesia care prior to laparoscopic adrenalectomy, our patient was more hemodynamically stable and experienced no significant perioperative complications after both procedures.

## Case presentation

An 84-year-old woman with a history of severe dementia, anxiety, hypertension, type 2 DM, and known aortic stenosis presented to the emergency department in February 2020 following multiple ground level falls without loss of consciousness and right knee pain. During workup, the patient was persistently hypertensive [systolic max = 209, diastolic max = 160] with shortness of breath, wheezing, an electrocardiogram re-demonstrating the history of left bundle branch block (LBBB), and aortic stenosis. Chest x-ray showed possible pulmonary edema and bilateral pleural effusions. Subsequent transthoracic echocardiogram (TTE) showed severe aortic stenosis (aortic valve area of 0.53 cm^2^, peak velocity of 4.1 m/s, peak gradient of 66 mmHg, mean gradient of 35 mmHg, and hyperdynamic left ventricular function with an ejection fraction of 20–25%). Previous TTE revealed diastolic heart failure. CT scans revealed no aortic abnormality and a mass in the adrenal gland with suspicion of pheochromocytoma. Upon review of history, an unspecified adrenal mass was discovered incidentally on CT scan in August 2019. The mass was confirmed to be a pheochromocytoma by 24-hour urine study with significantly elevated plasma metanephrines and catecholamines. The patient was lost to follow up after initial pheochromocytoma finding.

The patient was admitted for treatment of hypertensive urgency and heart failure and started on IV furosemide and hydralazine in addition to continuing home medications (carvedilol, amlodipine, lisinopril). Oral phentolamine was started to counteract sympathomimetic response from her active pheochromocytoma. She developed confusion and transient altered mental status overnight with suspicion of delirium. Upon improvement of her blood pressure, diuresis was reduced and hydralazine was discontinued. However, her blood pressure was later found to be 220 systolic with an elevated heart rate of 130 bpm and an elevated blood glucose of 300 mg/dl. The patient no longer tolerated oral phentolamine and was transitioned to IV phentolamine. A repeat troponin revealed a mildly elevated level of 0.33 ng/mL. Due to the patient’s labile vitals, intravenous diltiazem, metoprolol, and a nitroglycerin drip were initiated.

Additionally, due to concern for diabetic ketoacidosis she was upgraded to the intensive care unit. The patient remained persistently hypertensive around 200 and tachycardic between 120-130 bpm. The initial plan was to perform a laparoscopic adrenalectomy due to the active pheochromocytoma. The patient was started on labetalol and low dose vasopressin with anticipation of hemodynamic rescue in the event of catecholamine-resistant vasoplegia after pheochromocytoma resection and dexmedetomidine for agitation. However, her vitals remained labile with recurrent paroxysmal supraventricular tachycardia. Due to the patient’s cardiovascular instability secondary to severe AS, there was concern for a high risk of mortality during her adrenalectomy. A suggestion to perform a TAVR prior to removal of pheochromocytoma was recommended to combat high mortality risk in pheochromocytoma removal.

Interdisciplinary discussion concluded TAVR was the safest operative course for management of this patient and was scheduled for the following week, upon stabilization of patient’s vitals, and consent from her next of kin. The patient underwent monitored anesthesia care (MAC) sedation with judicious incremental doses of fentanyl (25 mcg every ~ 13 minutes; 100mcg total) and midazolam (2 mg initially, followed by 1 mg; 4 mg total) and a background dexmedetomidine infusion (0.5 mcg/kg/hr. - 7.78 mL/hr. followed by 0.25 mcg/kg/hr. - 3.89 mL/hr.; 28.77mcg total). Valve placement was conducted using a 23 mm balloon-expandable Edwards SAPIEN 3 valve. Percutaneous access was obtained and sheath angiography using a 5-French guiding catheter was performed of the RFA and LFA revealing the sheath in the common femoral artery. A balloon tipped pacing wire was advanced via the venous sheath to the RV. Baseline gradients were obtained, peak/mean/LVedp: 37/30/30 mmHg. The SAPIEN 3 valve was delivered via the delivery sheath and was loaded onto its deployment balloon within the descending thoracic aorta using fluoroscopy and advanced across the native aortic valve without difficulty. The valve was deployed under rapid ventricular pacing using aortic root injections at 10 cc/sec for 10 cc using diluted contrast. The valve was positioned in a satisfactory position at − 1-2 mm. Immediate transthoracic echocardiograph at this point demonstrated no paravalvular regurgitation. The Edwards delivery system was removed and a pigtail catheter was replaced into the left ventricle over the stiff wire and hemodynamics were assessed. Final gradients revealed peak/mean/LVedp: 1/4/24 mmHg with no paravalvular leak. Final angiogram demonstrated no stenosis, leak, or dissection. Severe intraoperative hypertension was treated with clevidipine infusion (2 mg/hr. - 4 mL/hr.; 1 mg total) and hypotension was treated with small aliquots of vasopressin. Alpha-blockade was maintained with phentolamine mesylate (10 mg total). The patient had an uneventful postoperative course. Patient remained stable throughout hospital discharge and was scheduled for adrenalectomy for pheochromocytoma resection 2 weeks later.

Following a 2-week hospital course without complications, the patient was brought into the operating room for laparoscopic adrenalectomy. General anesthesia was induced with fentanyl (200 mcg), midazolam (2 mg), propofol (200 mg), and rocuronium (50 mg). A clevidipine infusion was used for control of mean arterial pressure. Severe hypertension was treated with clevidipine infusion (12.18 mg/hr), nitroglycerin boluses (100 mcg), and severe hypotension was treated with boluses of ephedrine (20 mg) and vasopressin (3 units). Alpha-blockade was maintained with phentolamine mesylate (4 mg). A phenylephrine infusion was given to maintain and support vascular tone. The patient was extubated and transferred to the post anesthesia care unit for monitoring. Subsequent hospital course was unremarkable without upgrade to ICU status. On postoperative day 5, the patient was tolerating a well diet and her pain was well controlled on PO pain medications. She was ambulating on her own, had regular autonomic function, and was discharged home.

## Discussion & Conclusion

Severe aortic stenosis in conjunction with pheochromocytoma is a rare and high-risk situation [5, 6]. While resection of a symptomatic pheochromocytoma is strongly recommended and life-saving, valve replacement in the setting of symptomatic aortic valve stenosis is also necessary [7, 8]. There have been studies where TAVR was considered prior to pheochromocytoma resection, but was not feasible due to aortic valve size readings and mean gradients [9, 10]. The severity of our patient’s aortic stenosis from her TTE made her a suitable candidate for a TAVR. Performing a TAVR prior to pheochromocytoma removal may have provided better hemodynamic stability prior to pheochromocytoma resection. A recent case report explored a similar clinical scenario where a younger 51-year-old male presented with severe aortic stenosis complicated by a symptomatic pheochromocytoma [11]. Aside from the different patient demographic, their patient had a significant history of acute alcohol withdrawal and their approach of performing a TAVR under general anesthesia first prior to an open adrenalectomy differed from this case report’s anesthesia plan. While it remains controversial whether to perform TAVR prior or after pheochromocytoma resection, we attribute the absence of significant complications experienced during and after the procedures largely to the prior TAVR deployment under MAC anesthesia. The decision to have the TAVR procedure done under MAC anesthesia was based on several factors. There was known evidence of the dangers of laryngoscopy, hyperstimulation, decreased systemic vascular resistance and preload using general anesthesia. Furthermore, the characteristics of our patient was a convincing factor to proceed with MAC. General anesthesia can be associated with increased mortality and strokes among patients with aortic stenosis. This is likely attributed to increased catecholamine requirements [12, 13]. Given the complicated nature of our patient’s pheochromocytoma and concomitant severe aortic stenosis, the decision to proceed with MAC sedation in a judicious and carefully titrated manner was made. Other studies that have compared the use of general anesthesia versus MAC among TAVR patients demonstrated that those under MAC had a significantly shorter ICU length of stay and had reduced procedural, operating room, and fluoroscopy time [14, 15]. Our case report demonstrated similar findings with our patient experiencing no significant postoperative complications and no ICU stay.

Preoperative optimization of patients with active pheochromocytomas involves the use of alpha-1 and beta-1 inhibition to mediate the symptoms of their excess catecholamines. However, knowing when they have reached optimization can be difficult due to the variability of pheochromocytoma presentation and the wide array of effects on different organ systems. Furthermore, management can vary based on each patient’s characteristics and comorbidities. The physiologic end-points of an optimized pre-operative management still remains unclear. Current arbitrary goals include: blood pressure control for a minimum of 3–5 days, SBP < 130 mmHg, DBP < 80 mmHg, heartrate < 60 beats/min, no evidence of orthostatic hypotension with blood pressure < 80/45 mmHg, no ST-T wave changes on ECG, and Hct < 45 [16]. However, patients should also be closely monitored for end-organ damage due to excessive catecholamine release with the heart being the most commonly affected organ. This makes the goals of optimization more difficult in a patient with cardiovascular compromise, as the case with our patient. Ultimately, the patient presentation, their individual factors, and a judicious clinical decision will drive a personalized and optimized set point of when they are permitted for surgery.

Severe aortic stenosis in the setting of a pheochromocytoma remains a high-risk situation requiring a multidisciplinary discussion to plan the safest treatment option for each patient. While it has become a recommended guideline to treat severe AS in patients with severe systemic diseases, the distinctive feature of our case was the anesthesia choice of monitored anesthesia care for a TAVR prior to a laparoscopic adrenalectomy. This case demonstrated the successful usage of TAVR under MAC, which reduced the patient’s hemodynamic instability from their pheochromocytoma complicated by severe aortic stenosis. This case report’s anesthetic approach and order of clinical decisions led to the patient ultimately experiencing no significant intraperioperative and postoperative complications following both procedures.

## Data Availability

All data generated and analyzed are included in this article.

## Abbreviations

TAVR: transcatheter aortic valve replacement
AS: aortic stenosis
LBBB: left bundle branch block
TTE: transthoracic echocardiogram
MAC: monitored anesthesia care
RFA: right femoral artery
LFA: left femoral artery
LVedp: left ventricular end-diastolic pressure
